# Dynamic Thermal Structure of Imported Fire Ant Mounds

**DOI:** 10.1673/031.008.3101

**Published:** 2008-04-14

**Authors:** James T. Vogt, Bradley Wallet, Steven Coy

**Affiliations:** ^1^ USDA, ARS Biological Control of Pests Research Unit, PO Box 67, Stoneville, MS 38776; ^2^ Automated Decisions, LLC, 821 W. Lindsey St., Norman, OK 73069

**Keywords:** Thermography, remote sensing, insolation, nest architecture, *Solenopsis invicta*, *Solenopsis richteri*

## Abstract

A study was undertaken to characterize surface temperatures of mounds of imported fire ant, *Solenopsis invicta* Buren (Hymenoptera: Formicidae) and *S. richteri* Forel, and their hybrid, as it relates to sun position and shape of the mounds, to better understand factors that affect absorption of solar radiation by the nest mound and to test feasibility of using thermal infrared imagery to remotely sense mounds. Mean mound surface temperature peaked shortly after solar noon and exceeded mean surface temperature of the surrounding surface. Temperature range for mounds and their surroundings peaked near solar noon, and the temperature range of the mound surface exceeded that of the surrounding area. The temperature difference between mounds and their surroundings peaked around solar noon and ranged from about 2 to 10°C. Quadratic trends relating temperature measurements to time of day (expressed as percentage of daylight hours from apparent sunrise to apparent sunset) explained 77 to 88% of the variation in the data. Mounds were asymmetrical, with the apex offset on average 81.5 ± 1.2 mm to the north of the average center. South facing aspects were about 20% larger than north facing aspects. Mound surface aspect and slope affected surface temperature; this affect was greatly influenced by time of day. Thermal infrared imagery was used to illustrate the effect of mound shape on surface temperature. These results indicate that the temperature differences between mounds and their surroundings are sufficient for detection using thermal infrared remote sensing, and predictable temporal changes in surface temperature may be useful for classifying mounds in images.

## Introduction

Many ant species construct underground nests by simply excavating soil and depositing it around the nest entrance, eventually forming a simple crater. Mound-building ants, however, use excavated soil to construct earthen mounds (true mounds) containing numerous channels and chambers (Wheeler 1910). True mounds are constructed by species in some 10 genera distributed within three subfamilies of ants, including members of the genus *Solenopsis* Westwood. As first suggested by Huber (1810) and Forel (1874), ant mounds are generally thought to function as solar collectors, allowing for greater temperature variation throughout the mound strata, and providing ant colonies with a range of temperatures suitable for different life stages. Since that time a number of researchers have investigated the characteristics and functions of true ant mounds, primarily those of the genus *Formica* Linné (e.g. Frouz 2000, Galle 1973, Horstmann and Schmid 1986).

Red and black imported fire ants (*Solenopsis invicta* Buren and *S. richteri* Forel) are mound-building ants that were accidentally introduced into the southeastern United States during the first half of the 20^th^ century (Buren 1972). A broad band of hybridization between the species exists from central Mississippi to central Georgia (Shoemaker et al. 1994). Red, black, and hybrid imported fire ants appear to have similar mound-building habits and are referred to herein as “fire ants”. Their stinging habits, large colonies, and ability to build large mounds are just a few of the reasons they are considered serious pests (reviewed by Vinson 1997). Fire ant mounds can be quite large, depending primarily upon the size of the colony. Colony size can exceed 250,000 individual ants (Tschinkel 1993), and mounds can exceed 60 L total above-ground volume (Vogt et al. 2004). Fire ant mounds have been the subject of relatively few studies on thermal dynamics and/or thermoregulation, despite their abundance and size.

Porter and Tschinkel (1993) investigated thermal preferences of fire ants, demonstrating that colonies presented with a range of temperatures segregate brood into areas with temperatures favoring optimal growth (∼31° C). Some workers (about 30% in well-fed colonies, 50–60% in food-limited colonies) chose areas of cooler temperature (∼25° C), presumably prolonging their lifespan while not occupied with various colony tasks. This predictable behavior in the laboratory suggests that fire ants take advantage of a temperature gradient in the mound, residing in different parts of the mound as temperatures change during the day. Observations in the field corroborate this, as the bulk of a colony is usually located just beneath the exterior surface of the mound on sunny, cool mornings (Porter, as cited in Hölldobler and Wilson 1990; Vogt, unpublished observations). The interior structure of fire ant mounds, with narrow, horizontal tunnels below the crust, vertical shafts and nodules extending downward though the grass root system, and vertical shafts extending through flattened chambers underground, appears to facilitate movement of the colony as conditions change (Cassill et al. 2002).

Green et al. 1999 demonstrated stratification of fire ant mound temperature at two depths, and noted that mounds heated up more quickly during the day than surrounding, undisturbed soil. Mounds also cooled more quickly in the evening. Penick (2005) demonstrated increased surface heating of fire ant mounds on the south side relative to the north side, and a significant decrease in temperature between 1 and 5 cm of depth. By changing the temperature profile of fire ant mounds through reverse heating (shading the south side and reflecting sunlight onto the north side) he was able to induce the colony to alter the usual placement of brood within the mound, demonstrating that the ants primarily track temperature and other factors are probably not responsible for colony movement. Other insects such as subterranean termites that seek out areas with temperatures favorable for their development may reside in portions of fire ant mounds (Shelton et al. 1999).

Recent efforts using aerial multispectral digital imagery for detection of imported fire ant mounds have met with some success, resulting in detection of over 70% of mounds in pasture during May (Vogt 2004). However, in more heterogeneous environments such as ball-and-burlap plant nurseries, multispectral imagery is not sufficient for mound detection (Vogt and Oliver 2006). Aerial thermal infrared imagery may be an effective tool for mound detection in landscapes where fire ant mounds lack the ring of vigorous vegetation at their periphery which is typical in pasture areas during certain times of the year. This ring of vegetation is typically present during the spring months and is useful for interpretation of photos of multispectral imagery (Green et al. 1977; Vogt 2004). Aerial detection of fire ant mounds using multispectral or thermal infrared imagery would provide a means of quantifying mounds in areas where access is limited and could potentially reduce sampling costs associated with ground sampling (travel and person-hours), especially when detecting mounds over very large areas for survey, research, or precision insecticide application purposes. For example, research aimed at understanding the effects of landscape and habitat on fire ant abundance would be greatly enhanced through aerial detection of mounds. Fast, cost-effective, and accurate assessment of fire ant density over a large area would allow researchers to examine spatial autocorrelation of fire ant mound densities and associated changes in habitat and landscape variables at different spatial scales without the time and effort involved in physically sampling hundreds or thousands of closely-spaced plots on the ground.

**Table 1.**
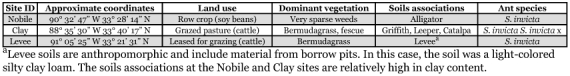
Study sites where thermal infrared images of *Solenopsis* spp. ant mounds were obtained.

A detailed analysis of daily changes in mound surface temperature is necessary for successful nest recognition in remote sensing efforts using airborne thermal infrared imagery. Based upon Green's (1999) and Penick's (2005) observations of mound temperature and our own preliminary data we hypothesized that the difference between the surface temperature of fire ant mounds and their surroundings would be sufficient for detection using thermal infrared imaging. We also hypothesized that the thermal center or weighted centroid of mounds in thermal images would move across the mound surface in a predictable manner, based upon the shape of the mound, sun elevation, and azimuth. Combined knowledge of temporal variation in mound surface temperature and anisotropy (direction) would benefit efforts to develop automated detection techniques for fire ant mounds in aerial thermal imagery, making quantification of mounds over large areas more feasible. An understanding of temporal variation in the surface temperature of mounds and their surroundings is necessary to optimize the timing of aerial data collection. Finally, a more detailed description of surface temperature than previously acquired may be useful to scientists interested in thermoregulation in fire ant colonies, and the role that the mound plays in those processes.

## Materials and Methods

### Data collection

Data were collected at three sites in Mississippi; site characteristics are summarized in Table 1. Sampling was conducted in the fall of 2005 (early October) and spring of 2006 (mid-April) during relatively dry periods with no rainfall for several consecutive days. Soil moisture in representative fire ant mounds was measured gravimetrically and was always < 5%. Wind speed was not measured during sampling but was generally low (< 5 m/sec) and the effect of wind and subsequent evaporative cooling on mound surface temperature was assumed to be low due to low soil moisture at the time of sampling. One day prior to sampling at a site, 5–8 active fire ant mounds were located and marked with forestry flags. Mounds were close enough to one another so that all mounds could be sampled within a 10 min time period. Mound width, length, and height were measured. A total of 16, 11, and 14 mounds were sampled at the Clay, Levee, and Nobile sites, respectively.

Thermal IR data were collected using a FLIR (www.flir.com) Thermacam ES thermal infrared camera with a 160 × 120 pixel microbolometer Focal Plane Array, a spectral range of 7.5–13 µm, and thermal sensitivity of ±0.12° C at 25° C. True color images were obtained using a Canon (www.canon.com)EOS 10D 6.5 megapixel digital camera. Thermal infrared temperature data were collected using an Omegascope (www.omega.com) OS531 infrared thermometer (accuracy of ±1.7° C), and kinetic temperatures were measured using a digital thermometer and surface thermocouple. Images were manipulated and analyzed in Environment for Visualizing Images (ENVI) + Interactive Data Language (IDL) (Research Systems, Inc., www.itt-vss.com) and ArcGIS 9.1 (ESRI, Redlands, www.esri.com).

Initially, the thermal camera was mounted on a tripod extended to a height of 2.5 m, pointing straight down at the ground for a horizontal field of view of about 1.1 m. In 2006 a new camera mount was constructed of PVC to raise the camera height to 3.7 m, resulting in a horizontal field of view of about 1.7 m. Once a mound was centered in the LCD display of the camera, the locations of the tripod or PVC mount legs were marked with large (3 cm dia) washers on the ground to minimize set-up time for images the following day. Three dental plaster (Castone) blocks (60 cm2) were placed next to each mound, one painted flat black (east side of mound), one painted satin white (west side of mound), and one unpainted (buff colored) (north side of mound). Care was taken to insure that the castone blocks fit within the images. Sampling generally commenced approximately 30 min to 1 h before apparent sunrise on the following day. At that time, and every 1 h until near or after apparent sunset, a thermal image was obtained of each mound. Surface temperature of the castone blocks, the approximate center of the mound, and the bare soil spot were determined using the infrared thermometer and recorded for each image. Ambient temperature about 1 m above ground level in the shade was recorded for each sampling time. Thermal images (jpg) were scaled for temperature in ENVI assuming a linear relationship between temperature (°C) and screen value in the 8-bit grayscale images. An IDL program was written to accomplish this, using the surface temperature of a pair of castone blocks as references. In preliminary laboratory experiments, the default emissivity setting on the infrared thermometer (0.95) yielded temperature measurements for mound soil and our castone targets that were similar to measured kinetic temperature. Pixel values in the resulting images showed close agreement with measured temperatures.

Sunrise/sunset and sun elevation/azimuth data were obtained from the National Oceanic and Atmospheric Administration Sunrise/Sunset Calculator (http://www.srrb.noaa.gov/highlights/sunrise/sunrise.html). The percentage of the mound surface obscured by vegetation (primarily grass) growing within the mound was estimated using RGB images registered to the 3D data. Vegetation was classified using the maximum likelihood classifier in ENVI with the probability threshold set to none. The resulting raster was clipped to the dimensions of the mound shapefile to estimate the area covered by green vegetation.

Three-dimensional (3D) data were collected using a Polhemus Fast-Scan Cobra™ laser scanner. Prior to 3D data collection (but after collection of thermal IR data), excess vegetation was clipped from the mound surface. A small (about 5 cm long) wooden arrow was placed beside the mound pointing toward magnetic north. The transmitter for the 3D scanner was placed within a few cm of the mound and oriented so that the axes ran north-south and east-west, then it was carefully leveled by placing a circular bubble level on top and removing or adding soil underneath. The mound was shaded with a surveyor's umbrella or a small hunting blind. When necessary, talcum powder was applied to the mound surface to increase reflectance. Scan progress was monitored on a laptop computer to insure complete coverage; the number of scan lines varied from mound to mound, and scanning was stopped when the operator was satisfied that a sufficient number of data points were collected. The resulting three-dimensional point clouds of mounds on a 1 mm Cartesian coordinate system were imported into ArcGIS as shapefiles. Mound surfaces were interpolated using an inverse distance weighted (IDW) procedure, with a power of 0.5, and a variable search radius with 12 points. Lowered power resulted in a more realistic surface, reducing the effects of grass that remained after clipping. To further reduce the influence of grass stubble, the focal statistics tool in ArcGIS 9.1 was used to calculate the mean of a circular neighborhood in the raster with radius of 3 cells. The resulting raster was used to extract statistics.

**Figure 1.**
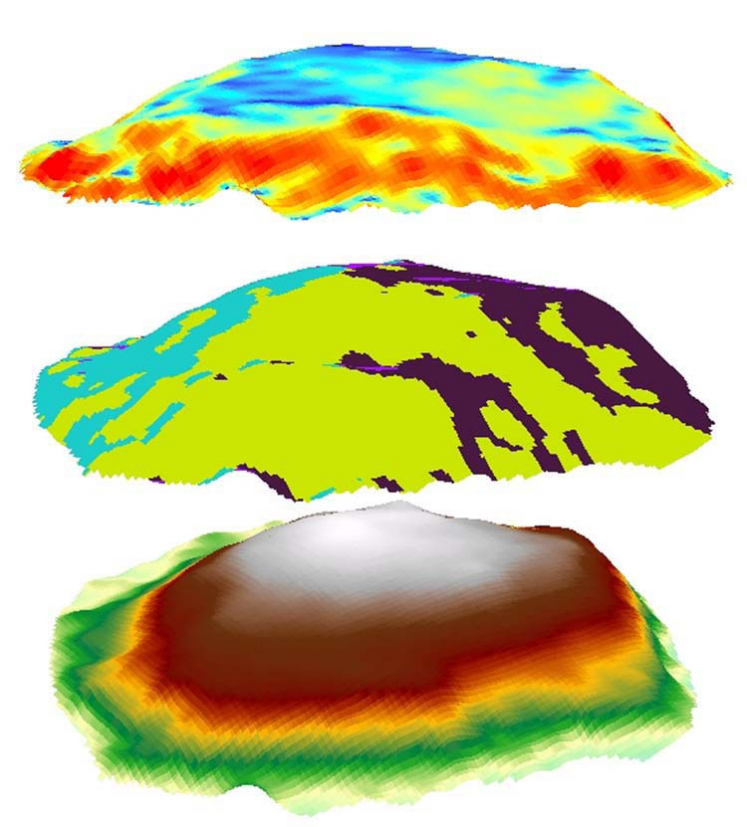
Data layers extracted from 3-dimensional point clouds of imported fire ant mounds; view is from the southeast. Slope (top layer) is scaled from gentle (blue) to steep (red). Aspect (middle layer) is classified as four equal intervals, and northeast (brown), southeast (yellow), and southwest (blue) are visible. Elevation (bottom layer) is displayed from low (light blue) to high (white).

The mean center of the mound feature was calculated. A new point shapefile was constructed corresponding to the mound apex. The 3D raster was used to calculate aspect, which was classified as four equal intervals of 0–90° (northeast), 91–180° (southeast), 181–270° (southwest), and 271–360° (northwest). Slope in degrees was also calculated and classified as gentle (0–25°), moderate (26–50°), or steep (> 50°). Physical mound characteristics were extracted from the data using 3D Analyst, Spatial Analyst, and Spatial Statistics tools in ArcGIS 9.1 (Figure 1).

### Data analysis

Analysis of variance (ANOVA) was used to examine mound characteristics: vegetation cover, volume, and location of the mound apex. ANOVA with Proc MIXED (Littell et al. 1996) was used to account for potential effects of location, sampling date, and their interaction. To further characterize mound shape, percentage of mound surface and slope in degrees were modeled as functions of aspect.

We first examined time effects on mound temperature, temperature of the surrounding area, and differences between mounds and their surroundings without taking mound shape into account. Statistics were extracted from thermal IR rasters using two zones defined by shapefiles. Mounds were carefully outlined by photointerpreting georeferenced RGB images and 3D models. An irregular area about 20 cm wide was outlined surrounding each mound shapefile to extract data on the area immediately surrounding the mound. Care was taken to avoid including castone reference targets in shapefiles.

A new variable was defined to standardize sampling times between date/location combinations, using sunrise/sunset data. Sampling time was expressed as the percentage of daylight hours from apparent sunrise (e.g., sunrise = 0%, solar noon = 50%, sunset = 100%; hereafter referred to as percentage day). Each sampling time was rounded to the nearest 8% day to maximize comparability between days while preserving the number of samples. Maximum, minimum, and mean temperatures were calculated, yielding mean temperature and range for mounds and their surroundings. Differences between mounds and their surroundings were also calculated for analysis.

**Table 2.**
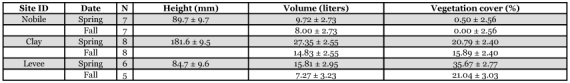
Estimated least squares means for above-ground volume, height, and vegetation cover of imported fire ant mounds at three study sites in Mississippi.

Four ANOVAs were performed. First, dependent variables (mean temperature, temperature range, etc.) were each modeled as a function of location, date, percentage day, percentage day^2^, and all possible interactions. Mound within location and date, and the three-way interaction between date, location, and percentage day were considered random effects. Second, to investigate possible effects of vegetation cover and mound volume (the total area underneath the mound surface in each 3D raster), data were averaged over each day, each dependent variable was modeled as a function of location, date, and location within date, and the independent variable of interest was included in the model as a covariate. Third, ground temperatures (mound surface and surrounding area) were modeled as functions of ambient temperature to determine whether they increased at the same rate as ambient temperature increased. Finally, slope, aspect, and all potential interactions were added to the model used in the first ANOVA for each location date combination to examine the effects of mound shape on surface temperature. Data are reported as mean ± SEM.

**Figure 2.**
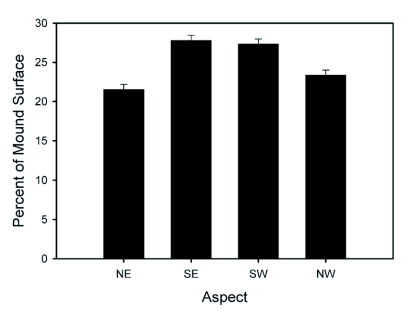
Mean percentage of fire ant mound surface by aspect for three Mississippi study sites. Error bars represent standard errors of the means.

**Table 3.**
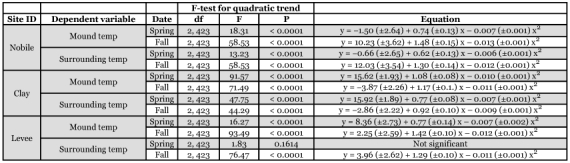
F-tests and regression coefficients for relationships between imported fire ant mound and surrounding area temperature measurements and time [percentage of day (x) between sunrise and sunset] at three Mississippi study sites.

Trends in temperature across the mound surface were visualized by creating an average raster for each time period and determining the average center of the mound weighted by temperature. For each location-date-time combination, each mound was registered to a single mound by using the mean center and the mound edge in each of the four cardinal directions as ground control points. This operation resulted in a series of mound images of approximately the same size and shape. The warped images were summed and divided by the number of mounds to yield an average thermal IR raster for each sampling time.

## Results

### Mound characteristics

Mound height, volume, and vegetation cover are summarized in Table 2. Vegetation cover on mounds was highly variable between locations (F_2, 35_ = 56.3; p < 0.0001) and dates (F_1, 35_ = 9.7; p = 0.0037), increasing from fall to spring with the exception of the Nobile site, where mounds had very little vegetation cover. Mean mound volume differed between locations (F_2, 35_ = 11.9; p < 0.0001) with a general increase from fall to spring (F_1, 35_ = 11.0; p = 0.0021). Mean mound height differed between locations (F_2, 32_ = 33.0; p < 0.0001), but not dates (p > 0.05). On average the apex of the mound was offset from north by 6.1 ± 1.5°. Distance from the mound center to the apex did not vary with location or date (p > 0.05) and ranged from 55 to 126 mm (mean = 81.5 ± 1.2 mm). There was a significant relationship between aspect and surface area (F_3, 159_ = 22.3; p < 0.0001) with a greater proportion of the mound facing south; the relationship between aspect and surface area was not affected by location or sampling date (p > 0.05) (Fig. 2). The slope of the mound surface (deg) was not influenced by aspect or sampling date, but varied strongly with location (F_2, 160_ = 112.9; p < 0.0001), ranging from 19 ± 0.7° at the Levee site to 31.8 ± 0.6° at the Clay site. Mounds can be subjectively categorized according to their state of repair; in this study, the state of repair varied widely between sampling sites. Mounds at the Levee site were generally in poor repair, while mounds at the Clay site were generally in good repair.

**Figure 3.**
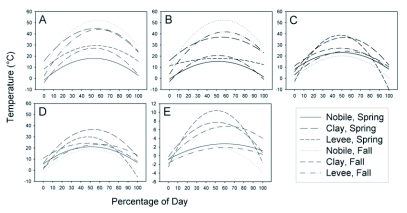
Quadratic trends for relationships between temperature (°C) and percentage of day from apparent sunrise to apparent sunset at three Mississippi study sites. A = mean mound temperature, B = mean surrounding area temperature, C = mean mound temperature range, D = mean surrounding area temperature range, E = mean temperature difference (mean mound temperature — mean surrounding area temperature).

**Table 4.**
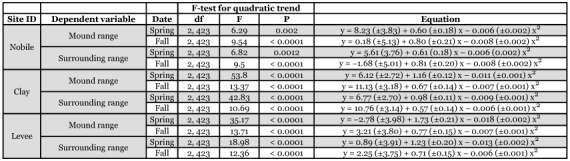
F-tests and regression coefficients for relationships between imported fire ant mound and surrounding area temperature range and time [percentage of day (x) between sunrise and sunset] at three Mississippi study sites.

### Mean temperatures

An examination of sums of squares indicated that time effects could be modeled as a quadratic trend. Location and sampling date explained significant proportions of the variation in mean mound temperature, surrounding temperature, temperature range, and the difference between mound and surrounding temperature; however, percentage day alone accounted for 77 to 88% of the variation in the models. Mean mound surface temperature was modeled as six separate quadratic trends corresponding to the different location-date combinations (Figure 3A). F-tests and regression coefficients for mound and surrounding area temperature relationships were obtained using contrast statements for each location-date environment, and are summarized in Table 3. Surface temperature of the area surrounding the mound varied similarly with time, but was up to about 10° C lower (Figure 3B); the only exception was the spring sampling date at the Levee site when temperature of the area surrounding the mounds remained relatively consistent throughout the day. Temperature of mounds and their surroundings generally peaked shortly after solar noon. Trends in temperature range of mounds (Fig. 3C) and their surroundings (Fig. 3D) were similar and were analyzed in the same way as described above. F-tests and regression coefficients are summarized in Table 4. Finally, the same analyses were applied to calculated differences between mean mound temperature and mean surrounding temperature to examine trends in the temperature difference over time (Figure 3E, Table 5).

**Table 5.**
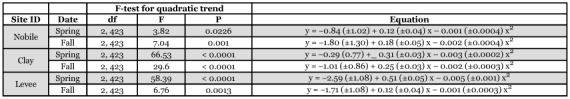
F-tests and regression coefficients for the difference between imported fire ant mound temperature and surrounding area temperature regressed over time [percentage of day (x) between sunrise and sunset] at three Mississippi study sites.

**Figure 4.**
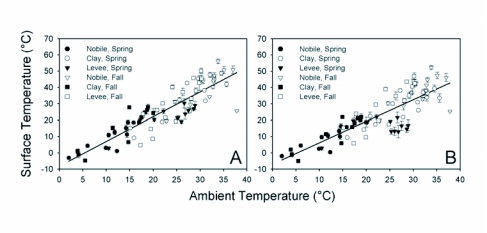
Relationships between ambient temperature and fire ant mound surface (A) and surrounding area (B) temperature at three Mississippi study sites. See text for equations.

Ambient air temperature was a good predictor of mound surface temperature (F_1, 535_ = 1612; p < 0.0001) and surrounding temperature (F_1, 535_ = 1149; p < 0.0001). Mound surface temperature increased about 15% more rapidly than surrounding temperature as ambient temperature increased (F_72, 923_ = 1.93; p < 0.0001). The effects of ambient temperature on mound surface temperature and surrounding area temperature are illustrated in Figures 4A and 4B, respectively. The equations relating mound and surrounding temperature to ambient temperature are Mound temperature = -7.74 (0.89) + 1.50 (0.04) Ambient temperature

Surrounding temperature = -6.57 (0.92) + 1.30 (0.04) - Ambient temperature

Interestingly, vegetation cover had a significant quadratic effect on mean mound temperature; however, the greatest change in predicted mean temperature within any location-date combination due to vegetation was < 4° C and vegetation effects were difficult to interpret due to low numbers of mounds analyzed. Neither mound volume nor mound height had any significant effect on mean mound temperature when added to the models as covariates (p > 0.05).

**Figure 5.**
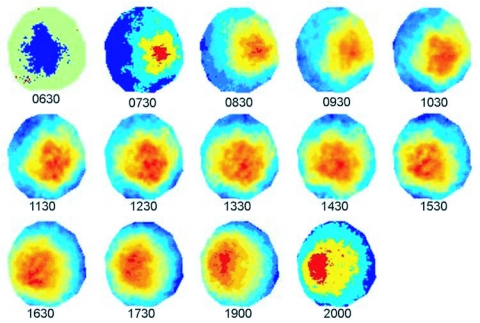
Time series of average thermal infrared rasters for eight imported fire ant mounds (Clay site, Spring). The “hot spot,” or area represented by red, moves across the mound surface in a predictable manner. Images are not temperature scaled.

### Effects of mound shape on surface temperature

While aspect and slope both influenced temperature when included in the models for quadratic trends over time, much higher F-values for their interactions with the linear and quadratic components of percentage day indicated that temperature differences among slopes and aspects were highly dependent upon movement of the sun (data not shown). Overall, the northern aspect of the mound tended to be about 6° C cooler than the southern aspect shortly after solar noon. A simple visual analysis using average rasters (see Materials and Methods) serves to illustrate the movement of the thermal center of the mound over time. As anticipated, the thermal center of the mound surface moved from east-southeast to west-southwest over the course of the day; Figure 5 is one example of a series of average rasters (n = 8 mounds) from the Clay site in spring.

**Figure 6.**
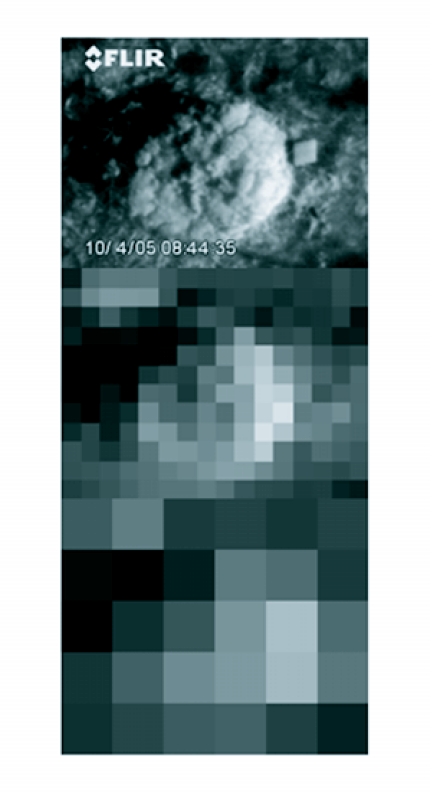
Thermal infrared image of a fire ant mound. Original image at 0.5 cm resolution (top); resampled image at 0.1 m resolution (center), and resampled image at 0.25 m resolution (bottom).

## Discussion

Temperature measurements in this study were modeled as a function of percentage day. These models could be used to predict temperature relations at different locations and during different seasons by correcting for sun position, so long as other conditions (soil moisture, cloud cover) remained constant. While surface temperature at any given point on the mound will depend upon solar angle of incidence, thermal history, and other variables such as soil type and surface texture, a model based on time of day is useful for considering timing of data acquisition, one of our broader goals. Maximum mound temperatures and surrounding area temperatures occurred shortly after solar noon (Figures 3A and 3B). Mound temperature was not measured during nighttime hours, but previous work by Penick (2005) demonstrated that mound temperatures decrease overnight to a minimum around sunrise the following day. While data were somewhat similar across date/time combinations in terms of the time at which maximum temperatures occurred, differences in the quadratic trends in terms of magnitude are probably due to differences in ambient temperature (the greatest mound temperatures occurred on the warmest days). Movement of fire ant castes and life stages within the mound was not investigated in this study; however, the data herein may be useful for making some predictions and testing hypotheses. For example, if fire ants move within the nest primarily in response to temperature (e.g., Penick, 2005), they should exhibit some east to west movement over the course of a day provided temperatures do not exceed about 32° C, at which point mortality of brood can occur (Porter, 1988). Mound surface temperatures should be very relevant to position of the ants within mounds, but more work is planned to build 3D models of mound temperature at varying depth.

The largest differences between mean mound temperature and mean surrounding temperature occurred within the location-date combinations with the highest percentage of vegetation cover on the mounds (Clay, spring; Levee, spring; Clay, fall) (Figure 3E). Vegetation is often distributed across the mound surface, but is thickest at the periphery and, while it is often more lush than surrounding vegetation, can be indicative of the general condition of vegetation in the surrounding area. Mounds with high vegetation cover still had >60% of the bare mound exposed to sunlight (Table 2) while the surrounding area was covered in vegetation. Those mounds with high vegetation cover were not hotter than other mounds (Figure 3A), so the increased difference in temperature for the location-date combinations listed above is probably due to shading of the surrounding soil by vegetation and/or transpiration. Those same location-date combinations also exhibited the greatest temperature range for mounds and their surrounding (Figures 3C and 3D). The greatest differences occurred around solar noon to early afternoon.

Fire ant mounds are asymmetrical, with the apex offset to the north. Asymmetry of fire ant mounds was previously noted by Hubbard and Cunningham (1977), Collins et al. 1993, and Vogt et al. 2004. In this study, the south-facing aspect of the mound was about 20% larger on average than the north-facing aspect (Fig. 2). To our knowledge, this is the first study to characterize mound shape by direct measurement of mound elevation across the entire mound surface followed by surface interpolation, which produces a more accurate estimate of aspect than visualizing the mound as four distinct quadrants or two distinct hemispheres. The unevenness of the mound surface, especially at the Levee site and to some degree at the Nobile site, resulted in some parts of the southeastern quadrant facing north, parts of the southwestern quadrant facing southeast, etc. These differences in mound repair were reflected in measurements of slope (see Results) and height (Table 2). Mounds in good repair tended to have a smoother surface with distinct boundaries between aspect classes and relatively steep slope. Mound repair and/or shape characteristics might also be related to soil type; the soils at the Clay and Nobile sites are clay-rich. Maximum slope is related to the properties of the construction material, and it is possible that slope at the Levee site was limited in comparison with slope of the mounds in heavier clay-rich soils. Generation of dense point clouds with the 3-D scanner allowed for accurate classification of aspect even for mounds that had an irregular or fractured surface. Overall trends in mound volume (Table 2) were similar to seasonal trends reported previously by Tschinkel (1993) and Vogt et al. 2004.

While state-of-the-art thermal infrared imaging equipment can detect very small temperature differences (< 1°C), both temperature range (a measure of variability) and temperature difference between the target and its surroundings will be important considerations when implementing a remote sensing program using thermal infrared imagery. Effectiveness of aerial remote sensing for detection of relatively small targets such as fire ant mounds will be dependent upon visual resolution of the data. Previous attempts to quantify mounds using airborne multispectral data were successful in detecting > 70% of mounds during the spring with 0.10 m resolution imagery (Vogt, 2004). At similar visual resolution, daily changes in the position of the thermal center of the mound may be important for constructing artificial templates for image classification and automatic object recognition. At coarser resolution, the overall difference between mean mound temperature and the surrounding area may be more critical. This point is illustrated in Figure 6. Additionally, if mean mound temperature is the more critical factor, data acquisition might be best undertaken around solar noon, but to take advantage of contrast between the warm and cool parts of the mound at finer resolution it might be best to acquire data early or late in the day. Experiments to test airborne thermal infrared imagery of different visual resolution for fire ant mound detection are ongoing.
